# Profile of caregivers of Alzheimer’s disease patients attended at a reference center for cognitive disorders

**DOI:** 10.1590/S1980-57642008DN10400015

**Published:** 2007

**Authors:** Marco Antonio Moscoso, Rita de Cássia Gomes Marques, Salma Rose Imanari Ribeiz, Lysandra dos Santos, Diana Moitinho Bezerra, Wilson Jacob Filho, Ricardo Nitrini, Cássio Machado de Campos Bottino

**Affiliations:** 1 CEREDIC (Reference Center for Cognitive Disorders); Clinicas Hospital of the School of Medicine of the University of São Paulo.; 2 PROTER (Old Age Research Group), Institute of Psychiatry - Clinicas Hospital of the School of Medicine of the University of São Paulo.; 3 Department of Internal Medicine, School of Medicine of the University of São Paulo.; 4 Department of Neurology, School of Medicine of the University of São Paulo

**Keywords:** Alzheimer’s disease, caregivers, burden, Zarit scale, neuropsychiatric symptoms, behavioral symptoms, psychotic symptoms, doença de Alzheimer, cuidador, sobrecarga, escala Zarit, sintomas neuropsiquiátricos, comportamento, sintomas psicóticos

## Abstract

This is a study on burden of caregivers of patients with Alzheimer’s disease attended at a Reference Center for Cognitive Disorders.

**Objective:**

To evaluate the profile and burden on caregivers of patients with Alzheimer’s disease attended at a Reference Center for Cognitive Disorders.

**Methods:**

We collected demographic information and data on the relationship with the patient from caregivers, and measured burden with the Zarit scale. The patients were evaluated with the following scales: the Cambridge Cognitive Test (CAMCOG); Mini Mental State Examination, the Neuropsychiatric Inventory for neuropsychiatry symptoms, and Functional Activities Questionnaire - FAQ for functional impairment.

**Results:**

Of the 31 caregivers, 77.4% were female, predominantly, and daughters, having a mean age of 58.6 years, educational level of 8.1 years, 70% of caregivers co-resided with the patient and 71% did not work. The mean time as a caregiver was 3 years. Twenty-seven percent of the caregivers presented mild to severe burden. The variables presenting significant association with caregiver burden were scores on the NPI and CAMCOG.

**Conclusion:**

The social demographic characteristics of the sample were similar to those of studies performed in other countries. The average time as a caregiver and the frequency of caregivers with mild to intense burden were lower than those reported in international studies. Neuropsychiatric symptoms and severity of cognitive decline were the main factors associated to burden in this sample of mostly mild to moderate demented AD patients. Further studies are necessary to verify whether the burden is indeed less intense in our milieu.

## Abstract (pt)

Trata-se de um estudo sobre a sobrecarga do cuidador dos pacientes com doença de Alzheimer atendidos em Centro de Referência em Distúrbios Cognitivos.

**Objetivo:**

Avaliar o perfil e a sobrecarga do cuidador dos pacientes com doença de Alzheimer atendidos em Centro de Referência em Distúrbios Cognitivos.

**Método:**

Foram obtidos dados demográficos do cuidador e sobrecarga de acordo com a Escala de Sobrecarga de Zarit. Os pacientes foram avaliados com os seguintes testes e escalas: Teste Cognitivo Cambridge (CAMCOG); Mini-Exame do Estado Mental; Inventário Neuropsiquiátrico (NPI), para a presença de sintomas neuropsiquiátricos; e o Questionário de Atividades Funcionais (QAF), de Pfeffer, para o comprometimento funcional.

**Resultados:**

Os 31 cuidadores eram predominantemente do sexo feminino, filhas, idade média 58,6 anos, escolaridade média de 8,1 anos; 70% dos cuidadores moravam com o paciente; 71% não trabalhavam. O tempo médio como cuidador foi de 3 anos. Vinte e sete por cento dos cuidadores apresentavam sobrecarga de intensidade leve a intensa. As variáveis que apresentaram associação significativa com a sobrecarga do cuidador foram os escores do NPI e do CAMCOG.

**Conclusão:**

As características sociodemográficas dos cuidadores foram semelhantes às de estudos feitos em outros países. Entretanto, sobrecarga foi menos freqüente do que em estudos internacionais. A intensidade dos transtornos neuropsiquiátricos e a gravidade do declínio cognitivo foram os principais fatores associados à sobrecarga nesta amostra constituída principalmente por pacientes com DA, com demência leve a moderada. Mais estudos são necessários para verificar se a sobrecarga do cuidador é menos intensa em nosso meio.

Alzheimer’s disease is a neurodegenerative illness characterized by a progressive decay of mental and physical capacity, increasing dependence and functional incapacity.^1^

According to the Alzheimer’s Association in the USA, 1996, seventy per cent of patients with AD are cared for at home by friends and relatives.

In the literature, there are several definitions for the term “caregiver”, but that proposed by Badia et al.^2^ embraces the caregiver’s most typical characteristics. These authors define the caregiver as a person who helps with the basic and daily instrumental life necessities for most of the time, without receiving pecuniary return for this activity.^2^

The concept of burden appeared in the early sixties but it was only in the early eighties that became the focus of health professionals. Burden can be defined as the sum of physical, psychological, social and financial problems which arise among members of the family or people who assist the ill elderly.^3^

Montgomery et al.^4^ define caregivers’ burden using the concept of objective burden which refers to practical problems associated with special care, such as continuous nursing care. However, subjective burden according to the same author is also called tension where this refers to the caregiver’s emotional reaction (e.g. low self esteem, anxiety and depression).^4^

Some epidemiological studies have demonstrated that psychiatric disorders, especially anxiety and mood disorders, are systematically more prevalent in families that take care of patients with Alzheimer’s disease than in the general population.^5^

Several studies refer to psychiatrics disorders as the most important consequences of being a caregiver. Besides these disorders, there are significant physical health repercussions, an increase in social isolation and aggravation of the economical situation.^6-8^

In our daily practice we observe that caregivers, after some time performing this activity, frequently present physical and psychiatric symptoms. This condition is known as The Caregiver Syndrome and is characterized by depression, tiredness, anxiety, sleep disorder, irritability, isolation, loneliness, self blame, memory and concentration difficulties, motivation loss, low self-esteem, besides cardiovascular, digestive, low immunity and metabolic alterations.^9-11^

It is important to point out that the caregivers, even the symptomatic, do not seek professional treatment and in most cases their condition goes undiagnosed.^12^

Therefore, a need has been identified to improve the qualitative evaluation of caregivers’ burden in order to diagnose and treat any mental and/or physical disorders that might arise.

To improve both evaluation and treatment of caregivers, there are appropriate instruments to evaluate the burden: the Caregiver’s Effort Index, described by Vitaliano et al.,^13^ and the Caregiver’s Burden Scale or Zarit Scale.^14^ The latter is the most used instrument to measure caregiver burden. The Zarit Scale is a scale that covers the caregiver’s health, psychological well being, finances, social life and relationship between caregiver and patient. The Brazilian version has been validated for use in Brazil by Scazufca.^15^

With regard to the setting of our studies, the only publication in Brazil to date on the impact of elderly with dementia on caregivers was conducted by Garrido and Menezes,^16^ who evaluated 49 people from a psycho geriatric service from São Paulo city.

## Objective

The purpose of this study is to evaluate the profile of caregivers of Alzheimer’s patients, observed at the reference center (“CEREDIC” - Clinicas Hospital Cognitive Reference Center/FMUSP), describing social demographic data such as gender, age, education level, professional activity, degree of similarity, and time as a caregiver, as well as to evaluate the burden of these caregivers according to the Zarit Scale and to investigate the association of these scores with results of cognitive tests, functional scales and caregiver characteristics.

## Methods

The sample consisted of 31 unpaid familial caregivers of Alzheimer’s patients from CEREDIC, as defined by Badia et al.^2^ This service has attended patients referred by the Psychiatric, Geriatric and Neurology outpatient units at Clinics Hospital/FMUSP with the purpose of investigating and diagnosing their cognitive disorders, since November 2003. The included patients are drawn from among the first 36 patients diagnosed with AD, according to DSM-IV^17^ and NINCDS-ADRDA^18^ criteria, in CEREDIC. AD patients were classified by dementia severity according to their MMSE scores: patients with 0 to 10 MMSE scores were considered severely demented, those scoring from 11 to 20 were considered moderately demented and with scores from 21 to 30, mildly demented.

The information obtained was gathered at the first interview with the relatives and patients. All subjects agreed to participate in the study. A questionnaire on the caregiver’s demographic data was applied including gender, age, educational level, professional activity, degree of similarity, whether they co-resided with the patient, and time spent as caregiver. The burden of this activity was measured using the Burden Scale by Zarit et al.^14^ and the severity of Burden was defined as scores higher than 46 points on the scale.

In addition, the following scales were used to aid patient diagnosis: CAMCOG (Cambridge Cognitive Test)^19^ and the MMSE (Mini-Mental State Exam)^20^ for cognitive evaluation, the NPI (Neuropsychiatry Inventory)^21^ to evaluate behavioral symptoms, and finally the FAQ (Functional Activities Questionnaire)^22^ to evaluate functional compromise. Patients' final diagnoses were established over the course of up to 3 visits to the CEREDIC.

Data analysis was performed with the *SPSS 14.0 for Windows* program. We present descriptive statistics, with mean and standard deviation. Comparison between the groups. divided according to burden level, was made using the Student t test. Regression analysis was performed with the score of the Zarit scale as the dependent variable, and age, gender, educational level, time as caregiver, MMSE, NPI, CAMCOG and FAQ as independent variables. To evaluate the linear regression model, the R squared change was used. The change in the R2 statistic is produced by adding or deleting an independent variable. Large R2 change associated indicates that the variable is a good predictor of the dependent variable. The significance level adopted for all tests was p<0.05.

## Results

Of the 31 AD patients included in the present study, 4 (12.9%) were classified as severely demented, 22 (71%) as moderately demented, and 5 (16.1%), as mildly demented. Considering the whole sample, 87.1% of the AD patients were classified as mild to moderately demented, according to their general cognitive status. Out of 31 caregivers of patients with Alzheimer’s disease, 77.4% were female. The mean age of the caregivers was 58.6 (±11.1) years, with mean schooling of 8.1 (±5.1) years. Sixty seven percent of the caregivers co-resided with the patient. Regarding professional activity, 29.0% held an outside job and 71.0% were dedicated fulltime to the care of the patient. In regard to the degree of kindred of the caregiver, the most frequent relationship was that of daughter at 61.3%, followed by husband at 16.1%, wife at 9.7%, sister at 6.5% and others at 6.4%. The average time as caregiver was 3.0 (±2.2) years. The scores from the tests and scales applied to the patients with Dementia are described in Table 1.

**Table 1:** Tests and scales applied in AD patients and caregivers.

	N	Mean	Std. Dev.	Minimum	Maximum
CAMCOG	31	52.44	13.63	25	79
NPI	31	34.97	25.64	4	117
MMSE	31	15.55	5.39	2	27
PFEFFER	31	17.45	8.45	3	30
ZARIT	31	31.77	16.95	6	72

CAMCOG: Cambridge Cognitive Test; NPI: Neuropsychiatric Inventory; MMSE: (Mini Mental State Exam); PFFEFER/FAQ: Functional Activities Questionnaire; ZARIT: Caregiver's Burden Scale or Zarit Scale.

Concerning level of burden measured by the Zarit scale, a total of 27% of caregivers presented a burden between mild and intense, corresponding to scores higher than 46 points. Table 2 below describes the level of caregiver burden according to the Zarit scale, comparing social and demographic variables of the caregivers along with tests and scales applied to the patients.

**Table 2:** Demographic and clinical characteristics of caregiver groups with and without burden (mean and standard deviation).

	Without Burden N=24	With Burden N=7	Student t test and p value
Age	58.33 (10.6)	59.71 (13.3)	t= 0.29 p=0.78
Educational level	7.50 (3.87)	10.14 (8.11)	t= -1.22 p=0.24
Timecaregiving (years)	2.50 (1.39)	4.50 (3.68)	t= -2.06 p=0.05
CAMCOG	51.96 (16.63)	33.29 (18.30)	t= 2.56 p=0.01
NPI	26.92 (22.17)	62.57 (33.24)	t= -3.33 p=0.00
MMSE	16.54 (4.90)	12.14 (6.01)	t= 1.99 p=0.06
PFFEFER	15.58 (8.28)	23.86 (5.79)	t= -2.46 p=0.02

CAMCOG: Cambridge Cognitive Test; NPI: Neuropsychiatric Inventory; MMSE: Mini Mental State Exam; PFFEFER: Functional Activities Questionnaire.

In the analysis of linear regression the only variables that demonstrated significant association with the Zarit scale were the NPI and the CAMCOG (Table 3).

**Table 3:** Coefficients of linear regression of ZARIT scale by NPI and CAMCOG.

	Beta	p value
NPI	0.414	0.012
CAMCOG	- 0.380	0.020

NPI: Neuropsychiatric Inventory; CAMCOG: Cambridge Cognitive Test.

Employing this model, we were able to explain 43.6% of the variation of scores from the Zarit scale. Significant association of the Zarit scale with other variables such as age, gender, educational level, time as caregiver, MMSE and FAQ by Pfeffer were not observed.

## Discussion

The results found in the present study are similar in many aspects with those found in several other studies on the subject. In the sample investigated, the 31 caregivers of patients were predominantly women (77.4%), a similar ratio to that observed by several earlier studies showing rates of between 63.4% and 87.0% of female caregivers.^2,8,16,23^ It is important to note that, in general, caregivers of the female gender present greater indexes of burden, depression and social isolation.^22,25,26,27^ In the present study, the mean age of the caregivers was 58.6 years, while studies by Carrasco et al.^7^ reported 56.0 years, 68.0 years^2^, 51.3 years^16^, 54.6 years^8^, and 59.0 years^23^. Therefore, the mean age of caregivers in several other studies, including ours, are similar falling within the sixth and the seventh decade of life. Hinton et al.^28^ noted that younger caregivers are more prone to stress and depression than older caregivers.

With regards to the degree of kindred, our study observed that of daughter as being the most frequent (61.3%), followed by husband. In the majority of studies reviewed including those by Schene,^29^ Carrasco et al.^7^ and Diaz et al.,^23^ wives were more frequent than husbands. Croog et al.^30^ evaluated the differences between wives and husbands as caregivers and verified that wives were more vulnerable to overload than husbands. Hypotheses for this difference include: the greater tendency of women to report, their physical and psychological symptoms, and that wives and husbands as caregivers differ with regard to the concept and role of caregiver. Finally, female caregivers can often have longer daily contact with the patient and can therefore be more affected due to persistent stress factors. Regarding physical, psychological and burden problems, studies suggested that women, and particularly daughters, are a high risk group as caregivers presenting higher vulnerability to these problems.

The average education level of our caregivers was 8.1 (±5.1) years of schooling. Garrido and Menezes^16^ stated that their caregivers had 8 or more years of education while Alonso et al.^8^ did not report mean education level in their study. They had only reported that 70.2% of the caregivers either had not attended school or had only attended primary school. However, Badia et al.^2^ stated that 39% of their caregivers had received schooling, and that 25.4% held either part-time or full-time jobs, while 25.4% were pensioners. On the other hand, the studies by Diaz et al.^23^ and Carrasco et al.^7^ did not include education level of the caregivers or whether they held jobs.

The education level of the caregivers in our sample was in line with that found in the Brazilian study by Garrido and Menezes.^16^ It might be valuable in future studies to evaluate caregivers with little schooling, in order to verify whether education level has any influence on the burden of the caregiver. Our regression analysis found no significant association between burden and education level. However, an earlier study showed higher levels of education and financial situation to be associated with lesser levels of stress.^31^ Another important finding on caregivers was that 67.7% of them co-resided with the patient, in contrast to the study of Garrido and Menezes^16^ which reported a rate of 81.6%. This difference may be related to the higher severity of the dementia syndrome, for the mean score on the MMSE was 12.2 in the cited study compared to 15.5 in the present sample, largely comprising (87.1%) patients with mild to moderate dementia. As the dementia syndrome aggravates, the patient becomes more dependent on the caregiver, leading to the caregiver residing full time with the patient. All studies observed that the physical and psychological symptoms of the caregiver, with regards to frequency and intensity, are closely linked to the time spent carrying out the activity of caregiver whereby the longer spent as caregiver, the greater the symptoms and burden evidenced.^23^

The literature reviewed suggested that caregiver burden was associated with many factors, including characteristics of the patient (severity and duration of dementia, behavioral problems and difficulties with daily activities), variables of the caregiver (age, tasks assigned to the caregiver, religious beliefs, ability to solve problems, ability to perceive illness) and variables related to the environment (financial resources, social support, quality of relationship in the past). Although, in the present sample, cognitive, functional impairment, as well as behavioral disturbances of the patients, have been cited as the variables most related to higher burden, many studies, including the present work, have not identified a simple relationship between severity of dementia and burden of the caregiver. Thus, the burden seems to be a product of the dynamic interaction between objective external stressors and subjective perceptions by the caregivers concerning the patients with dementia. Over the long term treatment of Dementia, the welfare of caregivers must be considered together with the treatment of the patient’s illness, not overlooking the fact that, frequently, caregivers face social, emotional, physical and financial losses, which become more and more significant as the patients’ illness progresses. Therefore, identifying factors that contribute to caregivers’ burden can pave the way for future studies that evaluate specific stress reducing interventions for the caregiver of patients with dementia and Alzheimer. Further studies are warranted to ascertain whether the lesser burden observed in our sample reflected the relatively low level of dementia severity, sample bias, or cultural characteristics of our population.
